# Long‐Term Performance and Safety of a Superficial HA Filler With Tri‐Hyal Technology on Different Facial Zones: Forehead, Cheeks, Crow's Feet, and Upper Lips

**DOI:** 10.1111/jocd.16565

**Published:** 2024-12-20

**Authors:** Hugues Cartier, Jean‐Jacques Deutsch, Frederic Braccini, Philippe Garcia, Agnès Ehlinger, Michel David, Frederic Loreto, Laurent Benadiba, Anne Grand‐Vincent, Elena Rumyantseva Mathey, Karim Nadra, Ferial Fanian

**Affiliations:** ^1^ Centre Médical Saint Jean Arras France; ^2^ Private Practice Paris France; ^3^ Private Practice Nice France; ^4^ Private Practice Thionville France; ^5^ Private Practice Metz France; ^6^ Alfa Medical Center Paris France; ^7^ Laboratoires FILL‐MED Paris France

**Keywords:** anti‐aging, dermal fillers, facial injections, fine lines, hyaluronic acid, skin rejuvenation

## Abstract

**Background:**

The function of injectable hyaluronic acid‐based fillers is to smooth dermal wrinkles formed during aging. The aim of this study is to evaluate the performance and safety of a dermal filler after its commercialization.

**Methods:**

In this context, an 18‐month prospective randomized single‐blind study for the efficacy and safety of ART FILLER Fine Lines (AFFL) was performed on the forehead, the upper lip, the cheek folds, and the crow's feet. The efficacy, the longevity, and the safety were evaluated after a single filler injection without any re‐touch injection. The persistence of the correction was evaluated at 3, 6, 9, 12, 15, and 18 months.

**Results:**

The observations performed on 196 subjects enrolled showed that AFFL injections induced a significant improvement in wrinkle correction for all the assessed areas. These effects were significant as soon as 3 weeks after injection and remained significant until 18 months. Furthermore, injections of AFFL were well tolerated and no severe event was recorded. The minor reported reactions were resolved within 3 weeks.

**Conclusion:**

AFFL with Tri‐Hyal technology is suitable and well tolerated for the treatment of superficial wrinkles without any irregularity or Tyndall effect and showed a prolonged efficacy for at least 18 months.

## 1 Introduction

Face aging is characterized by wrinkles appearance which is considered as major visible sign of aging. The histological causes of these visible skin changes are due to a decrease in fat pads mass and their localization [1], as well as skin dermis atrophy due to a significant decrease in extracellular matrix production. In addition to advanced age, several factors also accelerate the wrinkles appearance such as sun exposure [2, 3], smoking [4], or facial muscles contractions [5].

To minimize the wrinkles appearance and deepness, the use of dermal fillers is one of the most frequently used technique for face rejuvenation [6]. Hyaluronic acid (hyaluronan or HA) is one of the most commonly used dermal fillers, enabling the augmentation of soft tissues, the smoothing of wrinkles, and the treatment of superficial defects [7]. In addition to the conformational properties, HA injections have been shown to improve skin moisturizing [8], reduce the oxidative stress, stimulate the subcutaneous fat maintenance [9], and the production of extracellular matrix by the dermis [10]. In addition to its favorable outcomes on skin wrinkles, HA‐based fillers are considered a safe treatment and the adverse events are minor in most cases and occur mainly at the injection sites right after the injection procedures. These reactions rapidly resolve, and serious adverse events are rare [11].

Injections of HA‐based fillers showed efficient longevity to maintain tissue augmentation for more or less about 9 months [6]. This characteristic is permitted by the cross‐linking method by a chemical agent such as the 1,4‐butanediol diglycidyl ether (BDDE). Based on the molecular weight of HA in the formulation and the degree of crosslinking, the modified HA molecules transform into a highly viscous and insoluble gel suitable for wrinkle filling. These rheological properties of the HA gel determine its clinical efficacy and longevity within the skin. However, there are not many cross‐linked HA fillers which are supposed to be injected superficially with a good longevity without any Tyndall effect or surface irregularities.

ART FILLER Fine Lines has a unique combination of three sizes of hyaluronic acid chains (“Tri‐Hyal”) to give a specific option to modify three different cursors: ratio of very‐long chain to long chain, cross‐linking rate, and free HA concentration. This filler has a very soft texture and low volumizing properties to provide good spreading and tissue integration to smooth and plump delicate face areas while it has good cohesivity. ART FILLER Fine Lines also contains 0.3% lidocaine hydrochloride for its anesthetic properties. This concentration of lidocaine, commonly used in similar fillers, does not affect tolerance or effectiveness and significantly reduces pain during injections [12, 13].

Trevidic et al. reported the aesthetic benefits and the safety of ART FILLER Fine Lines injections for crow's feet in a comparative randomized clinical study [14]. However, there was no data available on the safety and performance of this product in the other zones. In this context, we carried out a prospective and non‐comparative post‐market study to evaluate the long‐term performance of ART FILLER Fine Lines for treating the superficial wrinkles found at the forehead, the upper lip, the cheek folds, and the crow's feet areas as well as its immediate and long‐term tolerance. The primary objective of this study was to measure the aesthetic improvement of the treated areas from baseline to 21 days post‐injection following a single filler application. Secondary objectives included evaluating the long‐term efficacy of wrinkle correction in various treated zones over 540 days (18 months) and assessing the filler's safety and tolerance.

## 2 Material and Methods

### 2.1 Test Items and Injection Procedure

**FIGURE 1:**
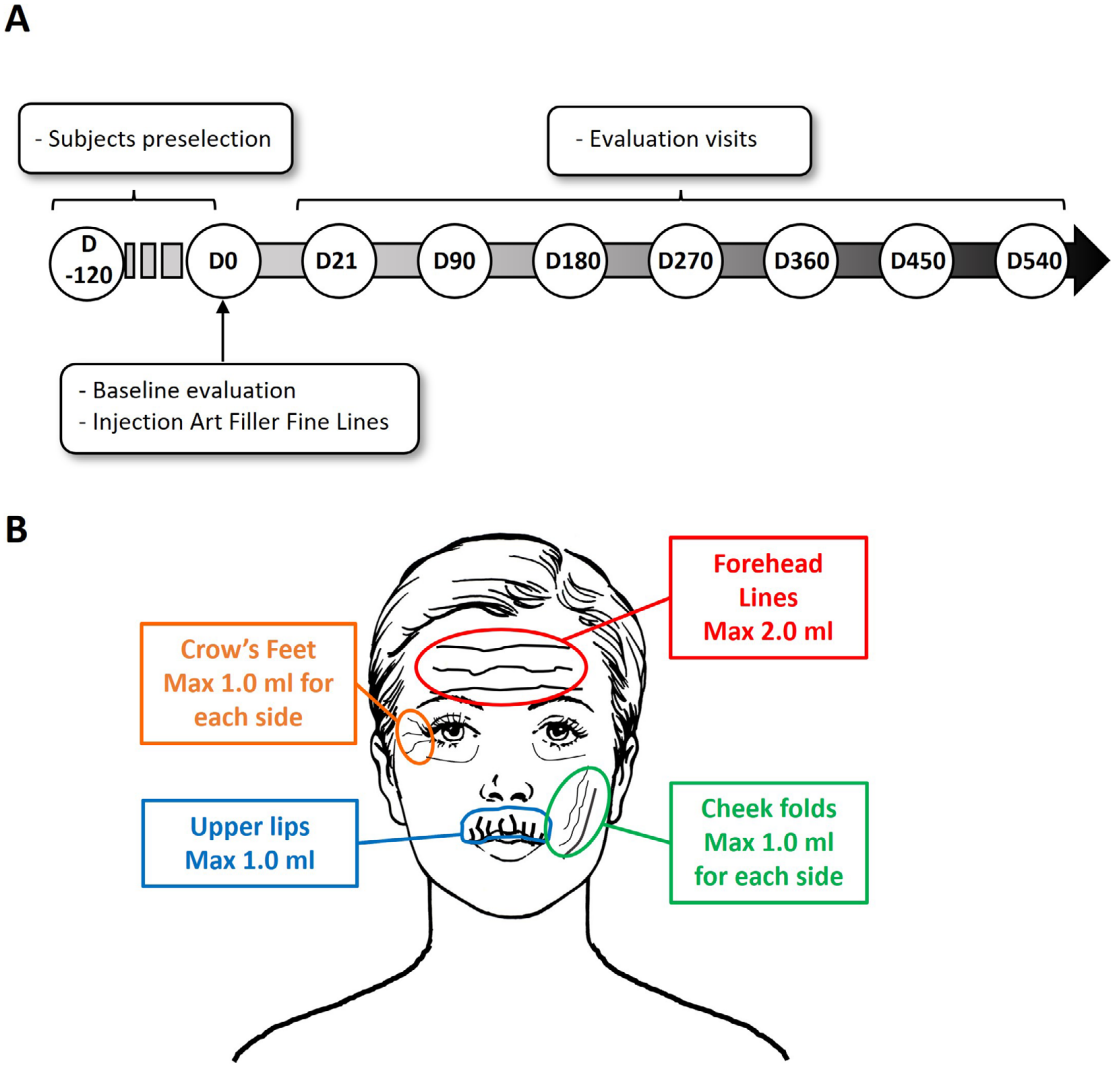
(A) Study flowchart over the 18 months period. After a first preselection visit between D120 and D0, the selected subjects were injected at D0. The follow‐up includes 7 evaluation visits carried out by the physician who performed the injections. (B) Anatomical zones of injection by the ART FILLER Fine Lines and the related maximal volumes.

ART FILLER Fine Lines is a crosslinked, hyaluronic acid of non‐animal origin, slowly absorbable over time, colorless, sterile, non‐pyrogenic, physiologically transparent, viscoelastic gel containing 0.3% by weight of lidocaine hydrochloride for its anesthetic properties. The amount of product injected varied according to the deficit to be corrected, but for this study the maximum volume of 2.0 mL was authorized to be injected for the forehead, 1.0 mL each side for the cheek folds, 1.0 mL each side for the crow's feet, and 1.0 mL for the upper lip according to the physician daily practice feedback (Figure 1B). The injection technique used for ART FILLER Fine Lines was intradermal injection with a classic needle of 30G/13 mm (TSK) which is present in the commercialized box. The process of injection was performed depending on the injected areas and the habits of physicians. No regional anesthesia through nerve blocks was authorized for this study for all zones in order to evaluate the pain score.

### 2.2 Population

The pre‐selection of subjects, conducted by the SYRES company or investigators between 120 and 7 days before the study (D‐120 to D‐7), adhered to specific inclusion and exclusion criteria. Participants were males or females aged 19 or older with Fitzpatrick Phototypes I–IV, and had a score of ≥ 1 on the Global Aesthetic Scale in at least one area. Eligible subjects received comprehensive information about the study protocol (Figure 1A). For each follow‐up visit (D21, D90, D180, D270, D360, D450, and D540), the per‐protocol population was defined by all the subjects seen at this visit and having an evaluation from the global aesthetical score. Safety population includes all subjects enrolled and for whom at least one injection of the studied product has been performed.

### 2.3 Evaluation Criteria

The Global Aesthetic Clinical Score (GACS) [15], was used for the main criteria of the study and was assessed at each visit for each injected area by the clinician in front of the subjects. This score system contains 7 grades scale, from 0 to 3 points with 0.5‐point interval (1 grade). The score 0 represented no wrinkles and score 3 represented deep wrinkles. In addition, according to photographic scale, the Bazin's visual scales for each specific area under investigation were used in order to double‐check the performance. These scales have been previously validated and published [16].

### 2.4 Ethical Approval

The protocol has been submitted to the French National Ethics Committee platform and finally has been validated by the Ile de France V CPP (St Antoine Hospital, 284 rue du Faubourg Saint Antoine, 75012 Paris) (People Protection Committee). This study was set up in the investigative centers only after obtaining the favorable opinion of this ethics committee on April 2, 2019.

### 2.5 Data Collection and Analysis

Laboratoires FILL‐MED provided the training for physicians and oversaw the implementation of this study involving the use of the devices. The evolution of the global aesthetic score between the baseline and the value of the selected day was calculated for each treated zone on the PP population. Mean score changes from Day 0 (D0) to follow‐up visits were computed, and their statistical significance was determined using a Wilcoxon test. Additionally, the change in the global aesthetic 7‐grade scale (score 0–3) from baseline (D0) to other time points was calculated for each treated zone in the PP population. The success rate was determined by the ratio of satisfactory responses at each time point. For the safety assessment, events were reported using the clinician's terminology, categorized by event type, and classified as local or general.

## 3 Results

### 3.1 Population and Procedure

In this prospective, non‐comparative study, a total of 196 subjects were enrolled (14 males and 182 females) with an average age of 53.7 years. Within this intention‐to‐treat population, 71% had no prior history of aesthetic treatment, while 21% had previously undergone HA injections. Subjects' weights were recorded before the injection and throughout the study to monitor their body mass index (BMI), which averaged 23.6 and remained stable. During the study, 21 subjects voluntarily withdrew before the final evaluation visit at D540. Injections were administered to a maximum of three different areas, including the forehead, upper lip, cheek folds, and crow's feet (Figure 1B). Efficacy was assessed at 3 weeks post‐injection, with the persistence of correction evaluated at 3, 6, 9, 12, 15, and 18 months (Figure 1A). Among the participants, 84 were injected in the forehead, 75 in the upper lip, 37 in the cheek folds, and 81 in the crow's feet. No touch‐up injections were allowed during the study. The mean injection volumes were as follows: 1.1 mL for the forehead, 0.8 mL for the upper lip, 1.6 mL for the cheek folds (both sides), and 0.9 mL for the crow's feet (both sides).

### 3.2 Evolution of the Global Aesthetic Clinical Score (GACS) and the Success Rate

**FIGURE 2:**
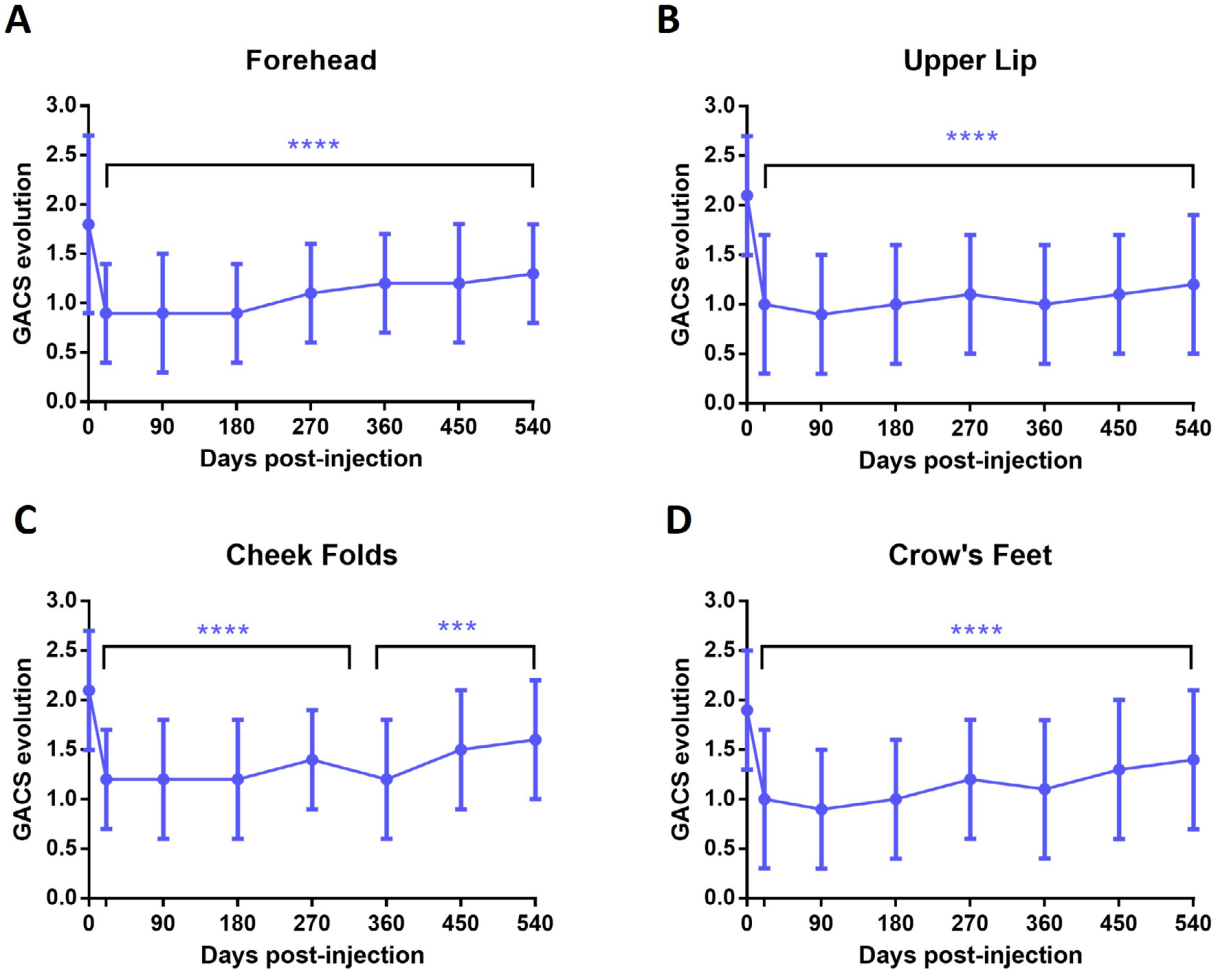
Remanence of the global aesthetic clinical scoring (GACS) between D0 and D540 on per protocol population for the forehead wrinkles (A), the upper lip wrinkles (B), the cheek folds (C), and the crow's feet (D). Mean ± SD, ****p < 0.0001, ***p < 0.001 compared to D0.

**FIGURE 3:**
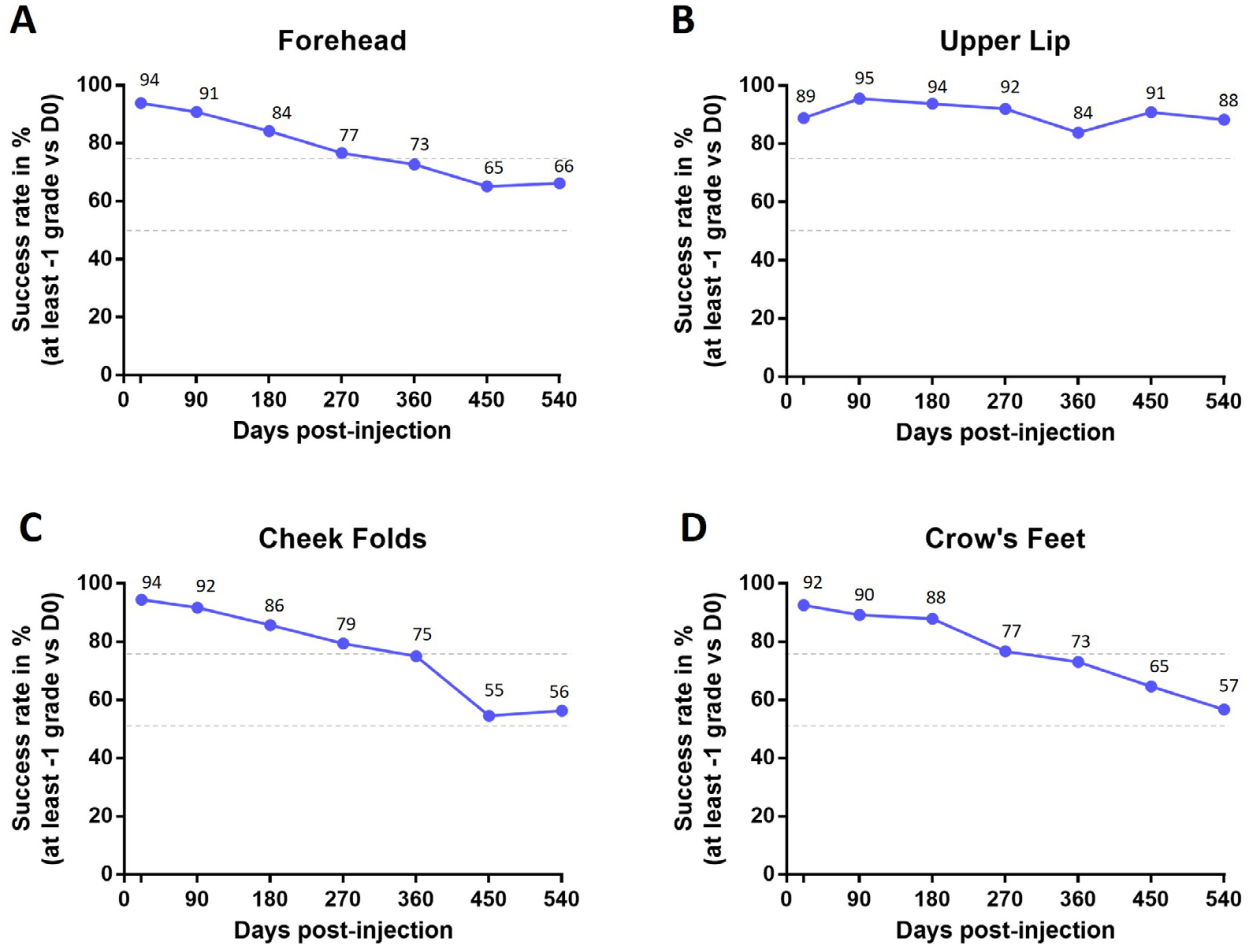
Evolution of the success rate represented by the percentage of subjects for whom the initial score was reduced from one grade. (A) Forehead wrinkles, (B) upper lip wrinkles, (C) cheek folds and (D) crow's feet. Percentages are calculated from the number of GACS values available for each visit. The upper and the lower dash lines represent 75% and 50% success respectively.

The primary objective of this study was to objectively measure the improvement of forehead wrinkles, upper lip wrinkles, cheek folds, and crow's feet after a single injection of ART FILLER Fine Lines, using the Global Aesthetic Clinical Scoring (GACS) system. The evolution of GACS scores from baseline to Day 21 was calculated for each treated area. A decrease of at least one grade (0.5 points) was defined as a satisfactory correction, in accordance with the criteria used in the study by Kopera et al. [15]. As illustrated in Figure 2, ART FILLER Fine Lines injection induced a significant improvement in GACS scores across all treated areas after 3 weeks. The forehead, cheek folds, and crow's feet showed an average score reduction of 0.9, while the upper lip showed a reduction of 1.1. These significant improvements were maintained throughout the study period, up to Day 540 (Figure 2A–C). Furthermore, the success rate at 18 months post‐injection was notable: 66% for the forehead, 88% for the upper lip, 56% for the cheek folds, and 57% for the crow's feet, for subjects whose initial GACS score improved by at least one grade (0.5 points) (Figure 3A–C).

### 3.3 Evaluation of the Correction Persistence Based on the Bazin Photographic Scales

**FIGURE 4:**
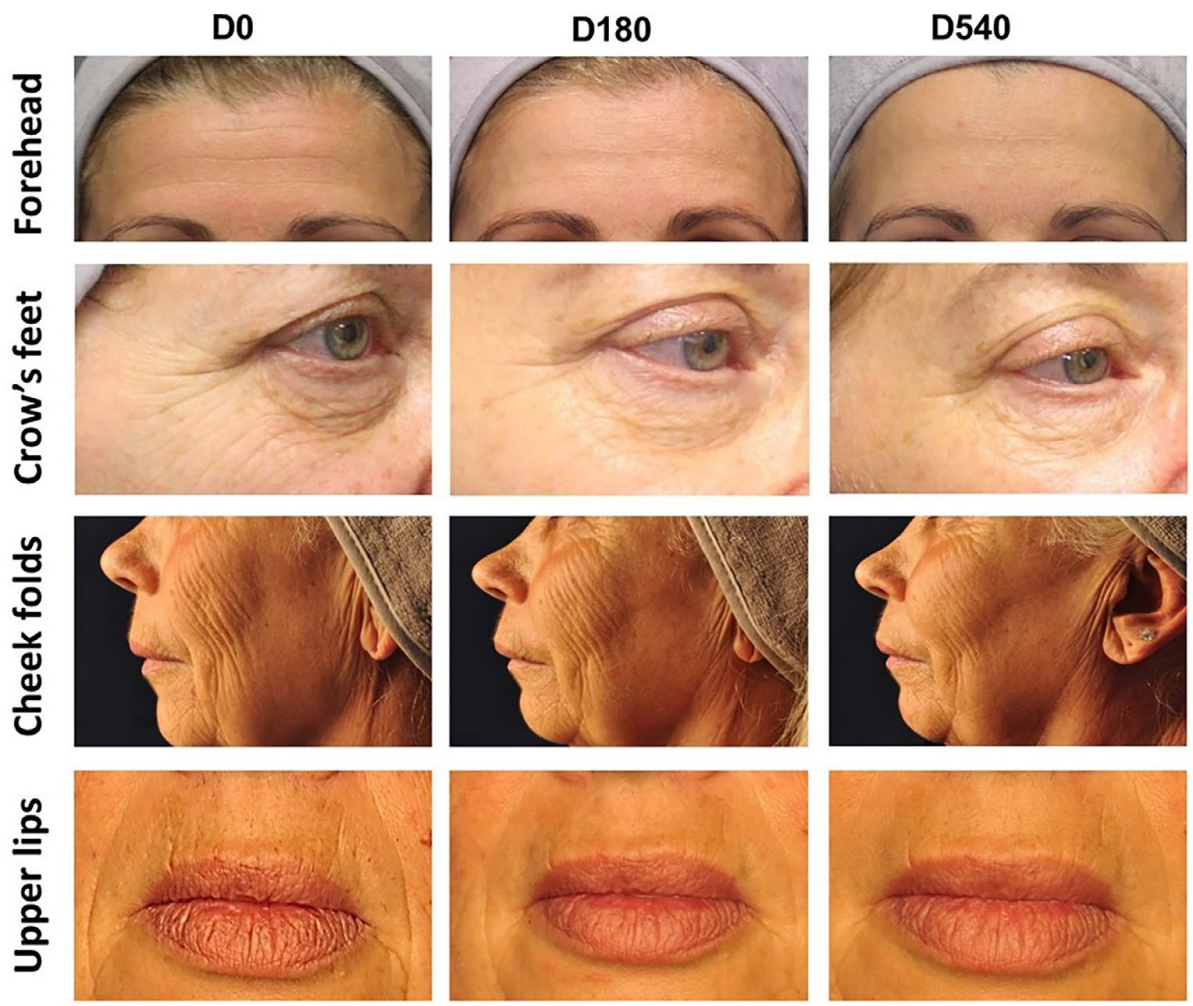
Representative images of 4 subjects depicting forehead, crow's feet cheek folds, and upper lip at baseline (D0), 6 months (D180), and 18 months (D540) post‐injection.

**FIGURE 5:**
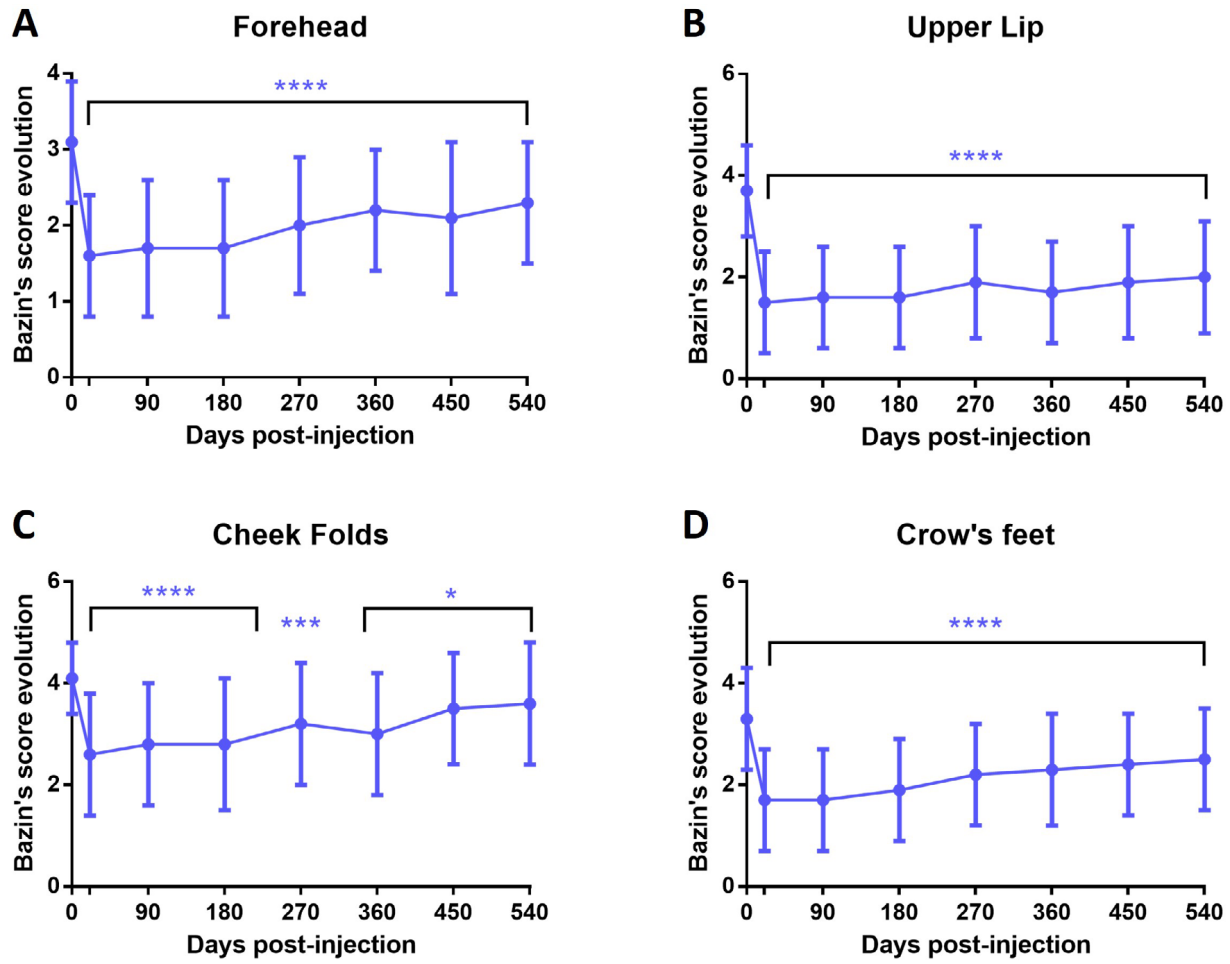
Evolution of the Bazin's clinical scoring (from photographical scales) between D0 and D540 on the per protocol population for the forehead (A), the upper lip (B), the cheek folds (C), and the crow's feet (D). Mean ± SD, ****p < 0.0001, ***p < 0.001, *p < 0.05 compared to D0.

To confirm the persistence of the observed corrections and the evolutions, each injected area was evaluated according to photographic zone‐specific scales. Representative photography at baseline, Day 180, and Day 540 of subjects treated in forehead, crow's feet, cheek folds, or upper lip are shown in Figure 4. The forehead Bazin's score was significantly decreased by −1.5 grade at 3 weeks post‐injection, represented by a success rate of 93.9%. This evolution was kept significant until day 540 (−0.8 with a success rate of 62%) (Figure 5A). The highest effect was observed in the upper lip area where the Bazin's score was reduced by −2.2 at 3 weeks with a success rate of 97.2% and remained significantly reduced over the study period to reach −1.7 at Day 540 with a success rate of 90.0% (Figure 5B). Similar evolutions were observed for the cheek folds and the crow's feet where the score was reduced after 3 weeks by −1.5 and −1.6, respectively, and −0.5 and −0.8 after 540 days (Figure 5C,D).

### 3.4 Assessment of the Global Aesthetic Improvement Scale (GAIS)

**FIGURE 6:**
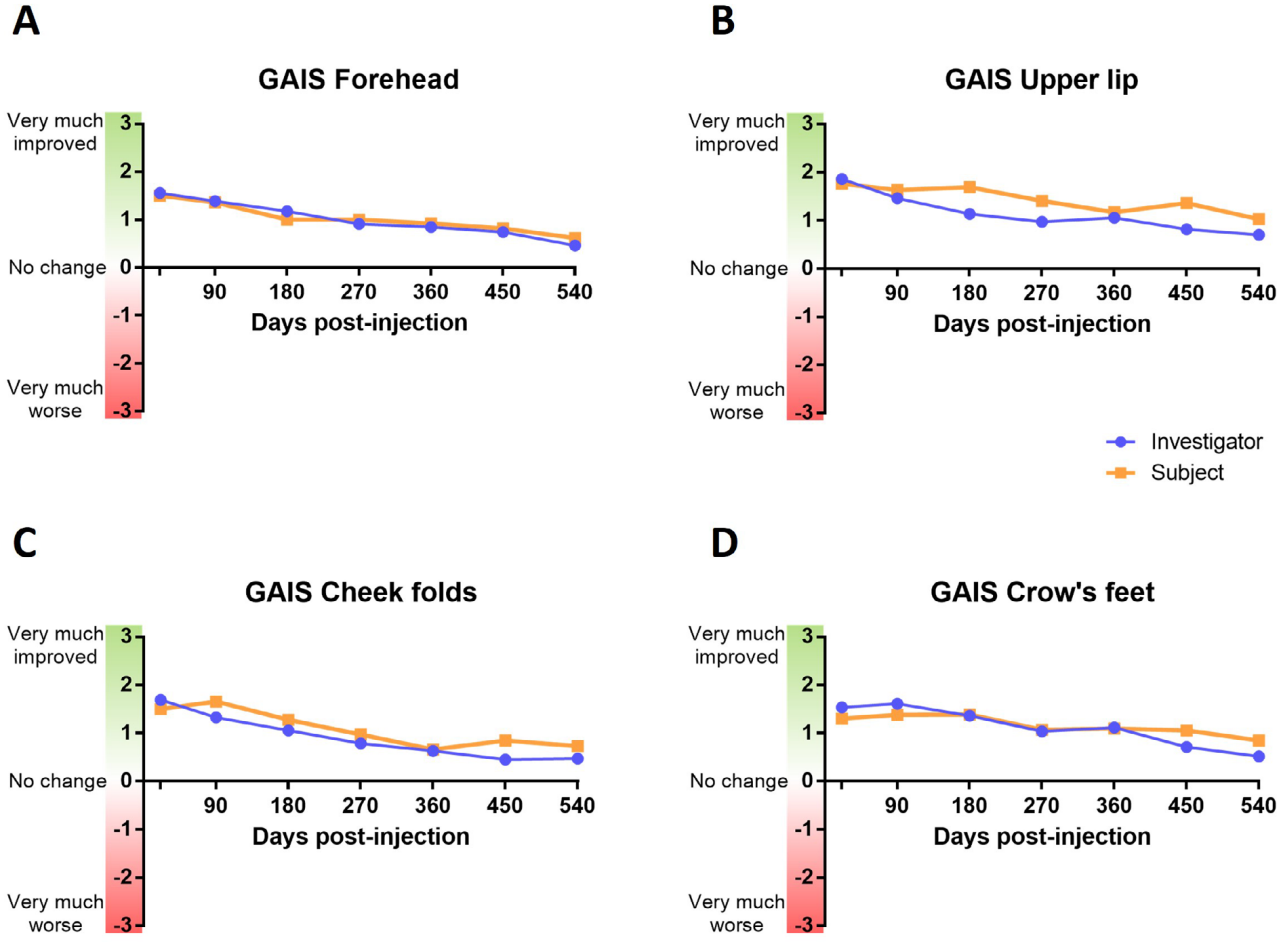
Global Aesthetic Improvement Scale assessed by the investigators or the subjects from D21 to D540 on the per protocol population for the forehead (A), the upper lip (B), the cheek folds (C), and the crow's feet (D). Data are mean calculated from the number of values available for each visit.

To confirm the favorable outcomes of the filler injections, both investigators’ and subjects’ satisfaction rates were assessed by the 7 levels Global Aesthetic Improvement Scale (GAIS) score from +3 for very much improved to −3 for very much worse. Globally, both investigators and subjects perceived an overall improvement for all the treated areas that persisted during the whole study (Figure 6A–D). The assessments performed by the investigators and by the subjects themselves were comparable. At 3 weeks post‐injection, between 90% and 97% of the subjects were improved whatever the treated area. This percentage slowly decreased to reach 41%–62% after 18 months, confirming the persistence of the benefits and its aesthetic outcomes.

### 3.5 Safety Assessment

**FIGURE 7:**
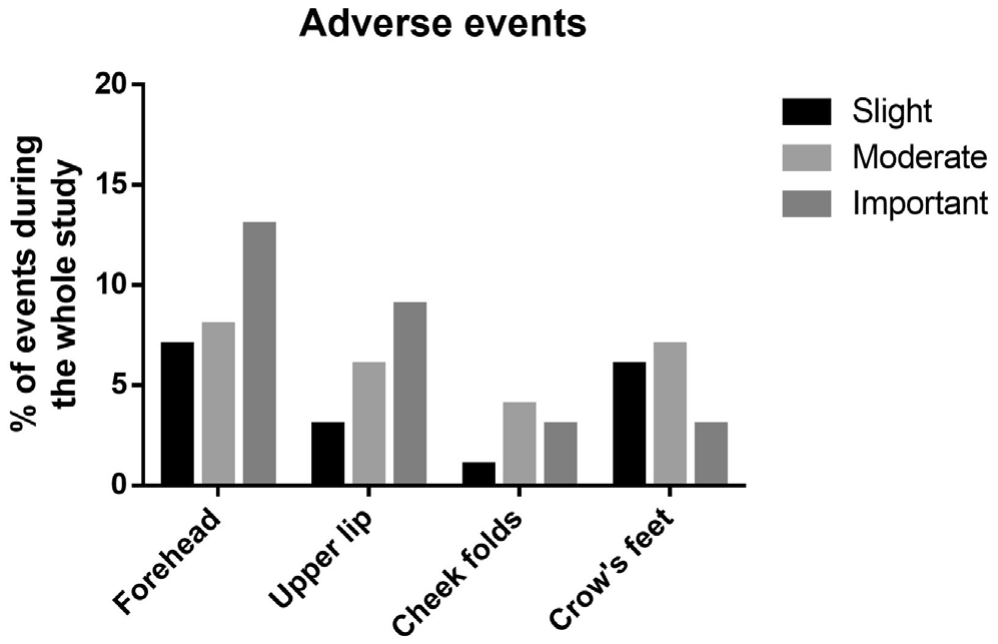
Percentage of the population that reported adverse events related to injections over the whole study period. The events were divided into 3 categories: slight, moderate, and important. No severe event was recorded during the study.

The appearance and sensitivity of the treated areas during and after injections were monitored. A total of 94 patients (48% of the total population) reported 269 adverse events throughout the study, encompassing all treated areas. Among these events, 77 (29%) were unrelated to the injections, 160 (59%) were possibly related, 25 (9%) were probably related, and 7 (3%) were doubtfully related. The most common adverse events included erythema, edema, and pain during injections (Table 1). As shown in Figure 7, these adverse events were classified from slight to severe. While no severe events were reported, 29% of subjects experienced adverse events after forehead injections, 17% after upper lip injections, 8% after cheek fold injections, and 16% after crow's feet injections. Overall, most of these events are commonly observed with HA filler procedures for facial volume correction, and the majority of local reactions resolved within 21 days post‐injection (Table 1).

**TABLE 1:** Frequency of the reactions reported on the forehead, upper lip, cheek folds, and crow's feet after injection at D0, D21, and D90. The percentage of reported signs was calculated from the number of values available for each visit.

	Forehead			Upper lip			Cheek folds			Crow's feet		
	D0	D21	D90	D0	D21	D90	D0	D21	D90	D0	D21	D90
Erythema	70%	—	—	48%	1%	—	49%	3%	—	53%	2%	—
Ecchymosis	22%	1%	—	6%	3%	—	19%	—	—	33%	1%	—
Hematoma	11%	—	—	7%	3%	—	6%	—	—	12%	—	—
Oedema	45%	—	—	44%	5%	—	13%	3%	—	36%	2%	—
Dyschromia	—	—	—	1%	—	—	—	—	—	—	—	—
Irregularity at palpation	18%	8%	6%	3%	3%	1%	8%	8%	—	11%	1%	—
Tyndall effect	—	2%	—	—	—	—	—	—	—	—	—	—
Over‐correction	4%	4%	—	4%	—	—	—	—	—	5%	2%	—
Pain at injection	58%	2%	—	47%	—	—	38%	—	—	28%	—	—
Pain after injection	8%	1%	—	7%	1%	—	11%	—	—	4%	1%	—

## 4 Discussion

This study reports the clinical benefits of ART FILLER Fine Lines in the reduction of fine superficial wrinkles found on the forehead, the upper lip, the cheek folds, and the crow's feet areas. The efficacy and the persistence of the aesthetic corrections were maintained until at least D540.

Exploratory analyses showed that the wrinkle correction by ART FILLER Fine Lines induced a persistent improvement in a high number of subjects injected in the upper lip. Indeed, 88% of the population showed a reduction of at least 1 grade in the global aesthetic clinical score 18 months after the injection. This parameter was also maintained significantly reduced but to a lower extent for subjects injected in the forehead (66%), cheek folds (56%), and crow's feet (57%). This difference between the upper lip and the other areas could be attributed to the skin characteristics and the wrinkles depth. Indeed, the contraction intensity and frequency of facial expression muscles could be less pronounced in the upper lip than other areas. A comparable effectiveness for the upper lip lines was described by Butterwick et al. in a previous study but during a shorter period of evaluation. In their study, the authors compared the efficacy of two other approved HA‐filers: Juvederm Ultra XC (Allergan) and Belotero Balance (Merz Aesthetics) [17]. As evidenced by the subject‐assessment effectiveness, 74% of subjects reported an improvement with ART FILLER Fine Lines in our study, 79% with Juvederm Ultra XC, and only 48% with Belotero Balance [17].

HA‐based fillers have shown persistence of their aesthetic benefits for a period between 12 and 14 months [6, 18, 19, 20, 21, 22]. Here, we report a significant persistence of the treatment efficacy for at least 18 months. One hypothesis about this efficient longevity observed with ART FILLER Fine Lines could be explained by its “Tri‐Hyal” technology. The specific combination of 3 types of hyaluronic acid (very‐long chain (3.4 MDalton), long chain (1.5 MDalton), and free HA (1.5 MDalton)), as well as their ratio could provide an appropriate condition for the resident fibroblasts to work in an hydrated extracellular matrix. In another hand, the cross‐linked portion of HA would persist in the dermis till 12 to 18 months and the Free HA will be slowly released from the formula. Furthermore, it was recently published that a “Tri‐Hyal” technology‐based filler could extend the subcutaneous adipocyte life and could be an excellent carrier of adipocyte cells to reconstruct and maintain the dimensions of volume loss [9].

Consistent with our prior findings [14], ART FILLER Fine Lines demonstrated excellent tolerance among subjects, with only minor side effects observed. Predominantly, reported reactions included transient pain immediately post‐injection, primarily manifesting as mild discomfort during injection, along with occasional instances of erythema or edema. The majority of these adverse events had resolved within 3 weeks post‐injection. These observed side effects align with the anticipated outcomes following HA filler injections [23, 24], affirming the safety profile of ART FILLER Fine Lines for treating the selected areas in this study.

In conclusion, our study confirms our previous observations on ART FILLER Fine Lines safety and tolerability in other face areas including the forehead, the upper lip, the cheek folds, and the crow's feet. This filler showed a sustained efficacy for at least 18 months in all assessed areas.

## 5 Conflicts of Interest

Ferial Fanian is currently the scientific Director and Karim Nadra was previously the employee of FILLMED Laboratories. No other conflict of the interest for the other authors. This study has been supported as a Post Market Clinical Follow Up by FILLMED Laboratories.

## Data Availability

The data that support the findings of this study are available from the corresponding author upon reasonable request.
